# Genomics-enabled analysis of specialized metabolism in bioenergy crops: current progress and challenges

**DOI:** 10.1093/synbio/ysaa005

**Published:** 2020-06-01

**Authors:** Kira Tiedge, Andrew Muchlinski, Philipp Zerbe

**Affiliations:** Department of Plant Biology, University of California-Davis, Davis, CA 95616, USA

**Keywords:** plant specialized metabolism, DNA synthesis, gene discovery, enzyme functional analysis, terpenoid metabolism

## Abstract

Plants produce a staggering diversity of specialized small molecule metabolites that play vital roles in mediating environmental interactions and stress adaptation. This chemical diversity derives from dynamic biosynthetic pathway networks that are often species-specific and operate under tight spatiotemporal and environmental control. A growing divide between demand and environmental challenges in food and bioenergy crop production has intensified research on these complex metabolite networks and their contribution to crop fitness. High-throughput omics technologies provide access to ever-increasing data resources for investigating plant metabolism. However, the efficiency of using such system-wide data to decode the gene and enzyme functions controlling specialized metabolism has remained limited; due largely to the recalcitrance of many plants to genetic approaches and the lack of ‘user-friendly’ biochemical tools for studying the diverse enzyme classes involved in specialized metabolism. With emphasis on terpenoid metabolism in the bioenergy crop switchgrass as an example, this review aims to illustrate current advances and challenges in the application of DNA synthesis and synthetic biology tools for accelerating the functional discovery of genes, enzymes and pathways in plant specialized metabolism. These technologies have accelerated knowledge development on the biosynthesis and physiological roles of diverse metabolite networks across many ecologically and economically important plant species and can provide resources for application to precision breeding and natural product metabolic engineering.

## 1. Introduction

**Figure 1.**
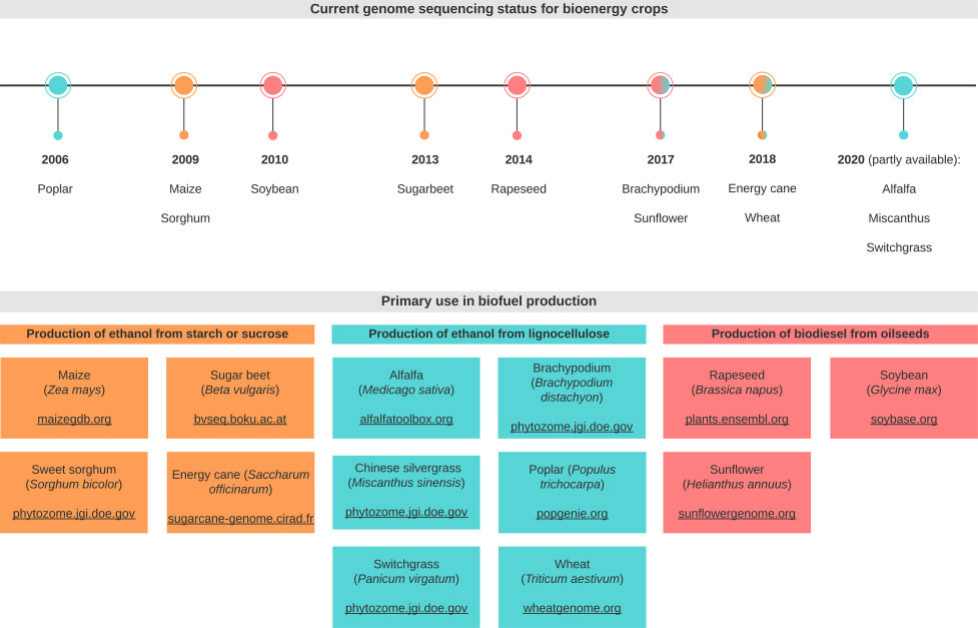
Overview of currently available genome resources for key bioenergy crops.

Meeting the needs of a growing world population while addressing urgent sustainability challenges has spurred intensive efforts in renewable energy production including biofuels (1, 2). In 2018, global biofuel production from plant-derived biomass reached an all-time high of over ∼150 billion liters, and the International Energy Agency projects production to increase by 25% over the next 5 years (3). Furthermore, U.S. Department of Energy assessments predict that 990–1150 M dry tons of plant biomass feedstock could be sustainably produced in the USA by 2030 (4), a level sufficient to supply more than a quarter of the current biofuel consumption, while maintaining food and feed production demands. First-generation agricultural feedstock crops such as maize, rapeseed, *Sorghum* and wheat, continue to serve as major resources for biofuel production (5) (Figure 1). In addition, cultivation of second-generation non-food model crops for biofuel production, including switchgrass, energy cane, poplar and *Miscanthus*, is becoming tractable to contribute to the needed production increase (5–10). But this development must address several key challenges of economic feasibility, balanced arable land use and a long-term sustainable environmental footprint (2). In the face of shifting environmental pressures that hamper plant fitness and cause agricultural yield losses (11), an important factor in advancing biofuel crop production is a deeper understanding of the genetic and biochemical mechanisms by which crops adapt to environmental perturbations. Such knowledge can provide key resources for agricultural optimization through precision breeding and targeted gene editing to generate crops that can withstand biotic and abiotic stressors, and enable scalable crop cultivation on marginal lands that do not compete with food production (12, 13).

Extremely diverse mixtures of small molecule natural products (termed secondary or specialized metabolites), such as terpenoids, phenylpropanoids, alkaloids, cyanogenic glycosides and glucosinolates, function as core mediators of plant–environment interactions (14–17). Among these metabolites, terpenoids are the largest class, comprising ∼40% of presently known plant natural products (17). Terpenoids are of essential importance to plant fitness due to their various functions as plant hormones, chemical defenses and signaling compounds in ecological interactions (14, 18). In addition, terpenoid bioproducts are of substantial economic value as food additives, therapeutics, cosmetics and biofuels (19–21). The latter includes several terpene hydrocarbons of high energy density, including farnesene, bisabolene or pinene, that are microbially manufactured as value-added alternatives to D2-biodiesel and high-performance aviation fuels (21, 22).

Their agricultural importance and broad use as bioproducts have driven a long-standing interest in developing robust strategies for the discovery and engineering of the genes, enzymes and pathways involved in plant specialized metabolism including terpenoids (12). Yet, progress in this field is hindered by the vast diversity of plant metabolism, limitations in pathway discovery tools and the often narrow taxonomic distribution of individual genes, enzymes and products that require analysis of the natural producer rather than established model systems (23). The post-genomic era has given rise to ever-advancing tools for generating genome, transcriptome and metabolome data that, in turn, have revolutionized the investigation of the biosynthetic and regulatory machineries controlling plant metabolism and how it impacts crop phenotypes and traits (12, 23, 24). In addition, developments in DNA synthesis and synthetic biology have made great strides toward establishing advanced approaches for the functional analysis of genes and enzymes involved in plant specialized metabolism (12, 25–28). Despite these advances, throughput in genetic and biochemical techniques for gene and enzyme functional studies lag behind the rapid increase in available omics data. As exemplified by the enzyme families of terpene synthases (TPSs) and cytochrome P450 monooxygenases (P450s), key terpenoid-diversifying enzymes, the functional diversity, dynamic regulation and taxonomic restriction of terpenoid and other specialized metabolite biosynthetic genes constrain reliable computational and functional annotation and necessitate empirical biochemical analyses (29–31). The latter then rely on the development of efficient platforms for plant transformation and/or the heterologous or cell-free expression and activity analysis of different enzyme classes.

With emphasis on specialized terpenoid metabolism in the bioenergy crop switchgrass (*Panicum virgatum* L.), this article highlights current progress and challenges in gene-to-function approaches. These approaches help to elucidate the molecular and biochemical foundations that govern the biosynthesis, diversity and function of specialized metabolic networks as an important foundation to agricultural crop optimization.

## 2. Omics-enabled pathway discovery in plant specialized metabolism

**Figure 2.**
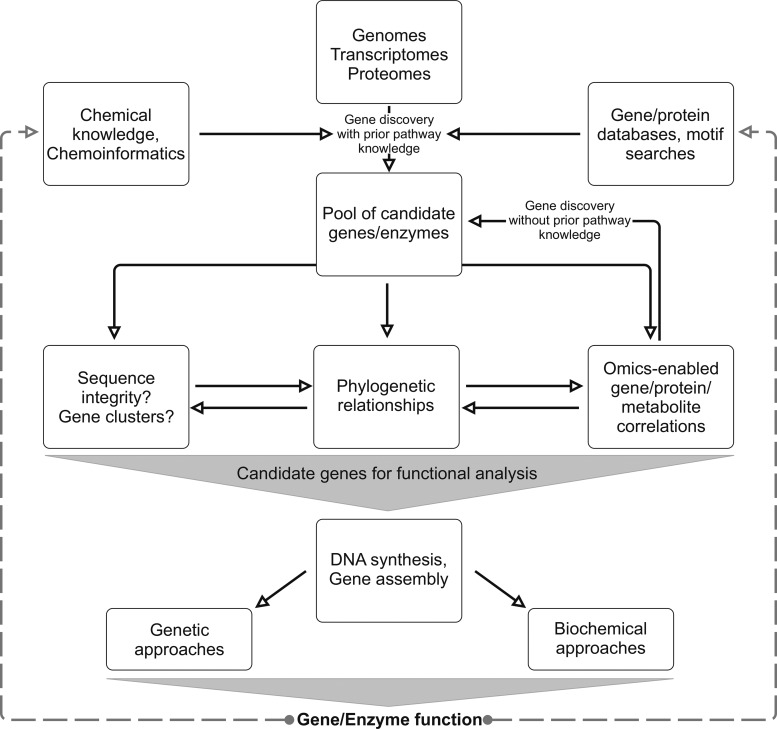
Scheme of possible customizable workflows for the omics-enabled discovery of gene candidates for subsequent functional characterization using genetic and/or biochemical approaches.

With sequencing and assembly platforms becoming faster and less expensive, the past decade has seen the release of numerous bioenergy crop genomes, including flagship biofuel feedstock and model crops such as maize, soybean, switchgrass, *Brachypodium distachyon* and poplar (32–41) (Figure 1). These genomic repositories form a broad foundation for pathway discovery, yet the road to unambiguously annotating gene functions can be rocky. As exemplified by the family of *P450s* that can comprise several hundred members in an individual species (30), gene families involved in specialized metabolism are often large and diverse as a result of repeated duplication of genes with ancestral functions in general metabolism, followed by sub- or neo-functionalization of duplicated genes (42, 43). Functional redundancy, catalytic promiscuity and emergence of new enzyme functions with as little as a single active site residue substitution as demonstrated, for example, for the TPS family (42, 44), curtail the reliability of sequence-based predictions of specialized gene and enzyme activities, thus requiring genetic and/or biochemical functional verification. In addition, computational gene annotation largely relies on comparison to well-established reference genomes, including *Arabidopsis thaliana*, rice and maize, which complicates the correct annotation of specialized gene functions that may often occur in only a single plant family or species. As a consequence, intensive efforts have focused on developing modular tools and strategies for reducing the pool of candidate genes and enzymes targeted for genetic and biochemical studies (Figure 2).

Where knowledge of the biosynthetic chemical reactions and enzyme classes relevant for different specialized metabolites exists and/or can be generated using chemoinformatic algorithms (26, 45, 46), such insights can be applied to querying genome and transcriptome inventories in a pathway/product-guided fashion using BLAST-enabled homology searches against gene- or protein-specific databases and/or signature protein motifs and domains to collect an initial pool of candidates (47–51). Numerous public gene, protein and motif databases are available for such approaches, including PhytoMine (https://phytozome.jgi.doe.gov/phytomine/begin.do), the Plant Metabolic Network (www.plantcyc.org), the Kyoto Encyclopedia of Genes and Genomes (KEGG, www.genome.jp/kegg) or Pfam (https://pfam.xfam.org/). The output of database searches should further be carefully curated to triage sequences with high likelihood of representing pseudogenes or misassembled and chimeric transcripts, a common occurrence especially among gene families with high degrees of sequence similarity such as the *TPS* gene family (31). In addition, although less widely distributed as compared to microbial natural product pathways, genomic co-localization of biosynthetic genes can provide important insights for candidate selection where specialized metabolic pathways can be organized as gene clusters. Such biosynthetic gene clusters have been demonstrated for several compound classes, including select pathways of diterpenoid, triterpenoid, benzoxazinoid, cyanogenic glycoside and alkaloid biosynthesis (52–55). The abundance and composition of such biosynthetic gene clusters are diverse across different species, ranging from 3 to 10 or more such clusters that may span genomic regions of 35 kb up to several hundred kilobytes containing three to six or more metabolic enzymes (52). Predominantly, these clustered genes will encode for enzymes catalyzing the first committed reactions channeling metabolites into specific specialized pathways as well as additional scaffolding or tailoring enzymes. For example, gene clusters containing multiple pairings of *TPS* and *P450* genes have been identified in diterpenoid and triterpenoid biosynthesis of several eudicot and monocot species, including biofuel model crops such as *Sorghum* and *B. distachyon* (53, 56). Likewise, in switchgrass co-localization of specialized class II and class I diterpene synthases occurs on chromosomes 3K and 3N, potentially indicating the presence of a diterpenoid-biosynthetic gene cluster (57). The mechanisms underlying the evolution of *TPS*/*P450* gene pairs appear to differ between eudicots and monocots and are not fully understood (53). Yet, discovery of operon-like triterpenoid gene clusters in *A. thaliana* demonstrated that, unlike horizontal gene transfer in bacteria, selection pressures likely drive clustering of defensive pathway genes in plants via gene duplications, neofunctionalization and genomic reorganization (58). Indeed, *TPS*/*P450* pairings occur more frequently across available sequenced plant genomes than would be expected from a random distribution, indicating the potential for co-regulation and co-expression that can be exploited for gene candidate triage (53). Continued advances in genetics, map-based cloning and bioinformatics approaches are expected to reveal biosynthetic gene clusters of specialized metabolisms across a wider range of plant genomes. Development of databases representing the defining parameters of plant biosynthetic gene clusters and bioinformatics tools that enable the mining of plant genomes for physically co-localized genes encoding enzymes of specialized metabolism (59), will provide broadly applicable tools for pathway discovery for species with available high-quality genomic data. In addition, experimental approaches that use operon-like, co-regulated regions of specific chromatin signatures associated with the repression or activation of biosynthetic gene clusters, more commonly known in animals and some fungi, have been demonstrated for screening plant genomes for co-localized, co-regulated gene clusters (60).

Complementary to patterns of co-regulation, and especially where no biosynthetic gene clusters are observed, functional insight can be further inferred from comparative phylogenetic analyses. Such approaches have become increasingly useful as more enzyme functions in plant specialized metabolism are discovered. Yet, these strategies have to be viewed with caution, since activity predictions can be complicated by the functional diversity and taxonomic rather than functional relatedness of specialized enzymes such as it is frequently observed in terpenoid metabolism (24, 29, 31). Combined sequence- and phylogeny-based gene discovery have been successfully employed to identify terpenoid-metabolic gene families in several monocot crops, including rice, wheat and maize (14, 61, 62). With the increasing availability of genomics resources, these methods can now be readily applied to a broader range of crops. For example, in the allotetraploid bioenergy crop switchgrass (cv. AP13), mining of genome and transcriptome inventories against a TPS-specific protein database (31) identified an expansive TPS family of more than 70 members (57, 63). Additional comparative sequence and phylogenetic analyses suggested a substantial divergence of the switchgrass TPS family, preventing functional annotations beyond a few more common TPS functions in gibberellin metabolism or those occurring in related monocot crops (57).

Considering the typically organ- or tissue-specific localization and often tight regulation of specialized metabolic pathways in plants, correlation of such sequence-based functional prediction approaches with genomics-, transcriptomics- and proteomics-enabled insights into transcript and protein abundance has become a powerful tool to identify pathway components that likely function together in individual tissues and under specific environmental stimuli (64, 65). Such approaches can further be integrated with advances in metabolomics and metabolite imaging technologies for identifying specific pathway components and their abundance *in planta* (49, 63, 66–68). For example, metabolite-guided pathway analysis recently led to the discovery of key *TPS* and *P450* genes for the biosynthesis of potent antimicrobial kauralexin diterpenoids in maize (62). Drawing on these pathway insights, gene expression correlation studies revealed additional gene functions, the products of which were later verified by metabolite profiling of maize tissues as previously unknown dolabralexin diterpenoids (69). Beyond co-expression analysis, where suitable diversity panels and plant populations are available, Genome-Wide Association Studies (GWAS) and Quantitative Trait Locus (QTL) analyses can guide the identification of genomic regions or individual genes with targeted physiological traits (70). For example, QTL studies led to the discovery of a steroid 5α-reductase that catalyzes desaturation reactions in kauralexin biosynthesis that were not predicted based on *a priori* pathway knowledge (62). Thus, such gene-metabolite and gene-trait correlation studies can be a facile tool for gene-guided pathway discovery where no or limited knowledge on the involved chemical reactions and corresponding biosynthetic enzyme classes exists.

Beyond pathway discovery comparative omics studies can guide the investigation of the physiological relevance of specialized metabolites and the corresponding biosynthetic pathways (23). For example, transcriptional analysis of switchgrass (cv. AP13) suggested a role of stress-inducible *TPS* genes in above- and below-ground herbivory defenses (63, 71). Metabolomics analyses further demonstrated the accumulation of steroidal saponin triterpenoids in several switchgrass varieties with possible functions in biotic stress defenses (72, 73). In addition, expression of specific *TPSs*, and corresponding accumulation terpene products, in response to drought and oxidative or ultraviolet stress, indicated possible roles of switchgrass terpenoids in abiotic stress tolerance (57).

## 3. Next-generation genetic and biochemical approaches accelerate functional studies

**Figure 3.**
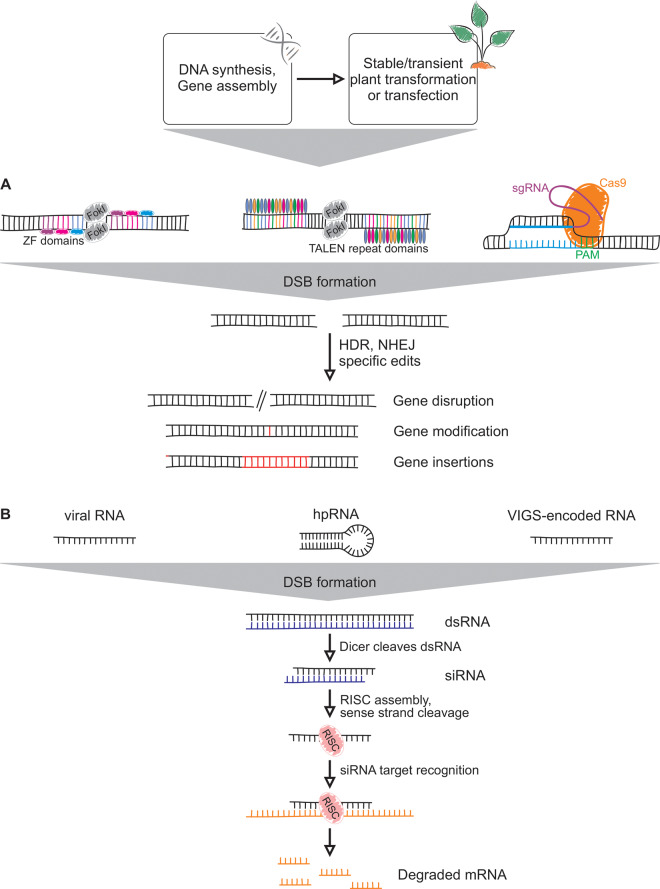
Schematic overview of possible genetic workflows for gene functional studies. (**A**) ZFN, TALEN, or CRISPR-Cas genome-editing nuclease approaches employ engineered non-specific nucleases fused to gene-specific DNA-binding domains to bind to a desired gene with high target specificity and induce double-strand breaks (DSB), followed by DNA repair via either non-homologous end joining (NHEJ) or homology-directed repair (HDR) to generate gene modifications that allow the analysis of the underlying gene function *in planta*. FokI, *Flavobacterium okeanokoites* type IIS restriction endonuclease; PAM, protospacer adjacent motif. (**B**) Gene silencing can exploit the formation of small interfering RNAs (siRNAs) by dicer-mediated cleavage of double-stranded RNA (dsRNA) generated from viral RNA, VIGS-derived RNA or hairpin RNA (hpRNA) that was transcribed from a transgene of interest. The antisense strand of the resulting siRNA guides an endonuclease (RNAi-silencing complex, RISC) to enable mRNA degradation in a gene-specific manner.

**Figure 4.**
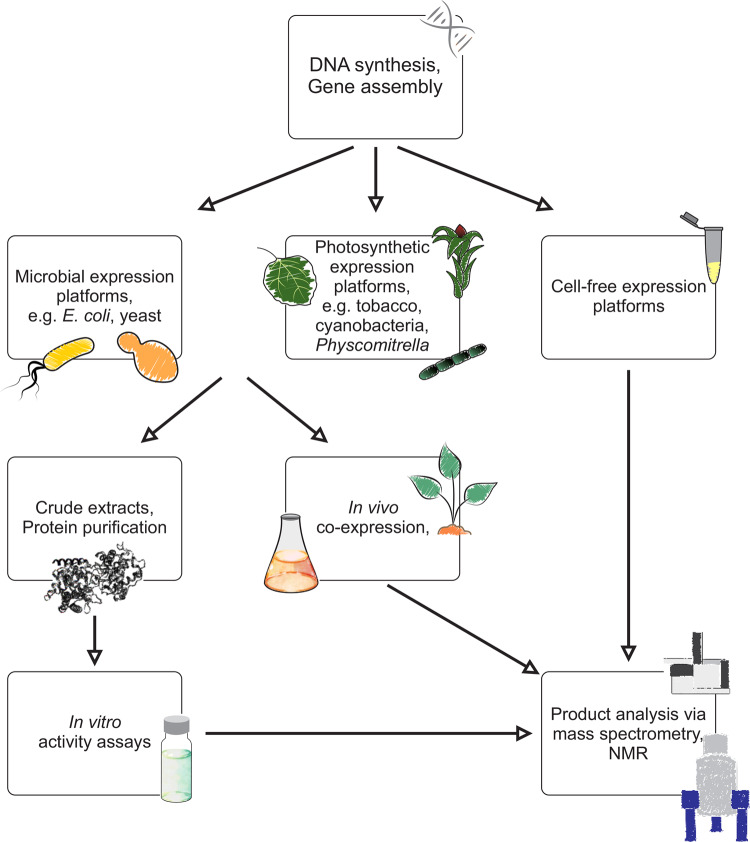
Schematic overview of possible biochemical workflows for enzyme functional studies. Following gene synthesis or cloning and assembly of gene constructs, numerous microbial, plant and cell-free platforms can be used for the heterologous expression of target enzymes. Enzyme functional studies can be conducted via protein purification and *in vitro* activity assays or using *in vivo* combinatorial enzyme expression strategies. Regardless of the chosen assay system, enzyme product identification typically involves liquid or gas chromatography-mass spectrometry (LC- or GC-MS) and/or nuclear magnetic resonance (NMR) analysis.

Downstream of candidate discovery, the verification of gene and enzyme functions requires robust genetic and biochemical approaches (74) (Figures 3 and 4). However, often low abundance of focal transcripts *in planta*, requirement for assay optimization for different enzyme classes and plant species and limited availability and knowledge of relevant substrates remain major factors constraining the throughput of these techniques. Alongside the enhanced quality and completeness of genome and transcriptome assemblies, DNA synthesis has become increasingly cost-effective for the rapid synthesis and modification of genes for functional analysis (75). Following the first gene synthesis of a yeast alanine tRNA in the early 1970s (76), today’s DNA synthesis platforms allow for the rapid, large-scale generation of even complex sequences at costs below 0.1 USD per base pair. In addition, DNA synthesis readily enables access to transcripts that are difficult to obtain via amplification, as well as gene modifications including codon-optimization, sequence modification and gene assembly, making genes substantially more amenable to a broader range of genetic and biochemical approaches (75, 77, 78).

Taking advantage of these DNA synthesis capacities, targeted gene modification/editing techniques, including nuclease-enabled gene modifications such as Transcription Activator-Like Effector Nucleases (TALEN), Zinc-Finger Nucleases (ZFN) and Clustered Regularly Interspaced Short Palindromic Repeats (CRISPR) approaches, as well as RNA-guided gene silencing techniques such as RNAi and virus-induced gene silencing (VIGS), can be used to identify gene functions *in planta* and without the need for *a priori* knowledge of pathway substrates and products (79–81) (Figure 3). Continued advances in the applicability of the CRISPR/Cas9 system, for example, by improving CRISPR/Cas9 catalytic specificity and efficiency by applying new generations of viral vectors (82), offers unprecedented opportunities for gene and genome editing. Furthermore, targeting of different genomic sites allows for simultaneously upregulating genes within a pathway while repressing others, resulting in modified genomes as well as transcriptomes (83). However, the complexity and high ploidy of many plant genomes, along with limitations in plant transformation and regeneration, continue to be a key bottleneck for the routine application of genetic gene-to-function studies; although, they are expected to continue to advance in coming years. In the case of switchgrass, variable typically high ploidy and self-incompatibility among diverse ecotypes poses a substantial impediment to genetic studies and crop optimization (84, 85). However, genetic approaches have been successfully employed in several instances, e.g. Xu *et al*. (86) employed an RNAi strategy to identify a 4-coumarate: CoA ligase relevant for reducing lignin formation. CRISPR/Cas9-enabled gene editing also has been successfully applied to switchgrass as exemplified by knock-down of a *UDP-Arabinomutase* gene that resulted in the accumulation of cell wall lignin (87). Likewise, CRISPR/Cas9-mediated loss of function of switchgrass *4-coumarate: coenzyme A ligase 1* (*Pv4CL1*) resulted in a reduced lignin content (88). Along with such gene-specific studies, the development of mutant collections, such as the *B. distachyon* T-DNA insertion collection and expansive maize mutant lines and diversity panels provide valuable resources expedite gene and pathway discovery (89–92). Continued optimization of plant transformation protocols will undoubtedly improve the applicability of genetic tools in switchgrass and other bioenergy crops (27, 93–95). For example, advances in *de novo* meristem induction techniques as demonstrated in select dicotyledonous species will help to overcome limitations in regenerating transgenic plants from tissue culture (27, 94). Complementary to stable plant transformation approaches, transient gene modifications can be applied to examine gene functions *in planta*. VIGS has been established as a powerful tool for transient gene function studies, since it is independent of plant regeneration protocols and defined genetic backgrounds (96–99). Availability of different VIGS vectors with a broad host range and high silencing efficiency and duration have proven useful for gene function studies associated with stress resistance in several crop plants (99–101).

Advances in synthetic biology and natural product metabolic engineering tools now also enable larger-scale protein biochemical analyses via improved protein expression and combinatorial pathway reconstruction in heterologous host systems, including yeast (*Saccharomyces cerevisiae*), *Escherichia coli*, *Nicotiana benthamiana*, *Physcomitrella patens* and cyanobacteria (21, 25, 102, 103) (Figure 4). Simplified gene modifications through DNA synthesis, such as codon optimization and altered signal peptides and compartmentalization, as well as advanced chassis for custom gene and promoter assemblies, have helped to overcome limitations in protein expression due to, for example, misfolding and feedback inhibition caused by lack of regulatory control in the heterologous host (103–106). However, while numerous robust protocols for heterologous expression, protein purification and *in vitro* assays have been established for many enzymes, insufficient soluble protein expression, low activity after translation and lack of relevant substrates remain key challenges. Advances in gene stacking strategies have become an invaluable tool for elucidating both known and novel biochemical pathways by means of combinatorial protein expression assays. This process has traditionally been reliant on introducing combinations of individual plasmids containing different pathway enzymes, which can present challenges due to high antibiotic loads and variable expression levels (107, 108). However, tools for simultaneous assembly of multiple target DNAs such as GoldenGate and related cloning methods have enabled more efficient reconstruction of pathways and enzyme combinations with additionally flexibly for tailoring expression levels using specific promoter, terminator and untranslated region (UTR) combinations (109, 110). A multiple chassis approach can also be used to facilitate discovery of natural products using non-conventional strains (103), in addition to a wider range of microbial and plant host platforms can be explored to optimize functional analyses (Figure 4). Microbial expression platforms offer the benefit of scalability and a wealth of molecular tools for gene and host optimization (25, 106, 111), but sufficient protein expression often remains a limiting factor for some enzymes such as the family of P450s. Additionally, plant-based systems have seen substantial advancement. In particular, *Agrobacterium*-mediated transient expression in *N. benthamiana* has become a routine system for enzyme functional studies, as is does not require feeding of carbon precursors, offers the natural compartmentalization for expressing plant proteins and naturally produces many key substrates for specialized metabolite biosynthesis (25, 112, 113). Current limitations of this plant expression system include an often higher metabolic background as compared to microbial systems, lack of endogenous precursor pathways for some natural product classes, and presence of endogenous enzymes and pathways that can interfere with introduced pathways through substrate competition and/or undesired metabolization of target compounds such as degradation or glycosylation (114). Bryophytes, especially *P. patens*, are also emerging as chassis for enzyme functional studies and pathway engineering, due to the availability of homologous recombination for precise genome-engineering and scalability in bioreactor platforms, yet require continued development of efficient synthetic biology tools along with a deeper metabolic understanding of these systems to enable more efficient and robust usage (115).

Next to *in vivo* expression systems, great strides have been made in developing cell-free expression systems for enzyme and pathway analysis and engineering, especially where heterologous expression is a limiting factor (116, 117). While membrane-associated proteins like P450s have been successfully produced in cell-free expression systems (118), misfolding and lack of activity after translation remain major challenges especially for membrane-bound proteins. The above approaches allow for the co-expression of genes of interest in different combinations to enable the efficient analysis of complex, modular pathways of plant specialized metabolism (25, 119–121). For example, combinatorial expression of identified TPS and P450 candidates in *E. coli* and *N. benthamiana* enabled the rapid discovery of modular terpenoid pathway networks in rice, wheat, maize and switchgrass (14, 57, 62, 69). Especially, where previously unknown enzyme functions are identified, the resulting products can, in turn, guide the discovery of metabolites and additional pathway genes *in planta* (69). In addition, pathway knowledge and new gene/protein functions deriving from the approaches outlined above, continuously expand the databases for improving the precision and efficiency of gene discovery and computational functional annotation (Figure 2).

## 4. Future directions

The biodiversity of specialized metabolism forms the foundation of the ability of plants to interact with and adapt to their environment with direct impact on plant fitness and agriculture. With an estimated number of more than 1 million plant compounds (122), this chemical repository offers an immense and largely untapped natural source to devise innovative strategies for crop trait discovery and improvement, as well as biomanufacturing plant-derived biofuels, medicines and other high-value natural products (12, 13, 21, 123). With our ever-increasing and cost-effective capacity for reading and writing nucleic acid sequences, unparalleled opportunities arise to unlock this biochemical potential. The examples discussed above highlight the power of integrating system-wide omics analyses with DNA synthesis and next-generation gene functional tools to decode the complex biosynthetic machinery controlling specialized metabolic networks in almost any organism. In this context, the continued development of higher throughput and more broadly applicable biochemical methods will be critical to accelerate the functional analysis of gene and protein candidates that result from genomics-enabled pathway discovery approaches. Advances in gene and enzyme modification through DNA synthesis and gene assembly can be integrated with new and improved microbial, plant and cell-free expression platforms to ultimately establish ‘user-friendly’ functional assay systems that can be readily applied to a broader range of enzymes and pathways. Further optimization of metabolomics, nuclear magnetic resonance and imaging tools can also be anticipated to continue to improve. This will simplify the often still cost- and time-intensive identification of enzyme products, and to precisely analyze the correlation of *in vitro* enzyme products with plant-, tissue- and cell-specific metabolite profiles (66, 67, 124). Likewise, gene editing and other targeted genetic tools must be further advanced to streamline the empirical verification of gene functions even in complex plant systems such as switchgrass (12, 123, 125). Alongside such approaches, facile techniques for transforming a broader range of plant species are urgently needed to apply genetic gene-function studies directly to the species where metabolites of interest are produced, and to improve the economic feasibility of optimizing crop traits (27). Driven by these transformative technologies, the rapid discovery of specialized genes, enzymes and pathways provides essential target genes for precision breeding and crop engineering. Furthermore, a comprehensive gene and enzyme catalog that can be translated into microbial and plant metabolic engineering platforms to produce high-value biofuels and other natural products, would be of societal benefit (21, 126, 127).
